# Next Day Subjective and Objective Recovery Indices Following Acute Low and High Training Loads in Academy Rugby Union Players

**DOI:** 10.3390/sports6020056

**Published:** 2018-06-15

**Authors:** Mark R. Noon, Rob S. James, Neil D. Clarke, Richard J. Taylor, C. Douglas Thake

**Affiliations:** 1 Faculty of Health and Life Sciences, Coventry University, Coventry CV1 5FB, UK; apx214@coventry.ac.uk (R.S.J.); ab1633@coventry.ac.uk (N.D.C.); d.thake@coventry.ac.uk (C.D.T.); 2 Department of Physical Education and Sports Studies, Newman University, Birmingham B32 3NT, UK; rtaylor.runner@googlemail.com

**Keywords:** athlete monitoring, athlete well-being, self-report questionnaire, performance

## Abstract

The aim of this study was to determine the sensitivity of selected subjective and objective monitoring assessments in detecting changes in group and individual responses to low and high load bouts of high intensity intermittent exercise. In a counterbalanced crossover design, Thirteen Academy Rugby Union players (mean ± SD: age: 18 ± 1 years) performed a low load (15 min) and a high load (90 min) bout of high intensity intermittent exercise (Loughborough Intermittent Shuttle Test) one week apart. Monitoring assessments were performed immediately prior to and 20 h following each trial. Subjective self-report Well-being Questionnaire (WQ) items showed small to large deteriorations following the high load compared to low load (*d* = 0.4–1.5, *p* = 0.03–0.57). A very large increase in resting HR (HR_rest_) (*d* = 2.1, *p* = 0.02), moderate decrease in heart rate variability (HRV) indices (*d* = 0.7, *p* = 0.04 and *d* = 0.7, *p* = 0.01 for the natural logarithm of the standard deviation of R-R intervals (ln SDNN) and the root square of the mean squared differences of successive R-R intervals (rMSSD), respectively) and no change in countermovement jump (*d* = 0.0, *p* = 0.97) were evident following the high load compared to low load. Individual WQ responses revealed 7/9, 7/9, 6/9, 6/9, 5/9, 3/9 and 1/9 participants reported deteriorations in recovery, sleep quality, motivation, muscle soreness, fatigue, stress and appetite, respectively, following the high load compared to low load. Individual analysis indicated a negative response following the high load compared to low load in HR_rest_, ln SDNN and ln rMSSD for 4/6, 2/6 and 1/6 participants, respectively. Selected WQ items detected group and individual responses to high load and low load highlighting their potential utility. However, objective assessments lacked the sensitivity to detect small individual changes.

## 1. Introduction

Subjective and objective assessments applied to monitor an athlete’s recovery status can assist coaches in effective training prescription, thus reducing the risk of non-functional overreaching (NFOR) alongside optimising the stimulus to promote training adaptation [1]. Accordingly, methods applied to monitor aspects of athlete recovery must be sensitive to changes in recovery status induced by different training loads. Self-reported well-being questionnaires have been proposed as valid measures to assess athlete responses to daily training and competition [2]. Previous studies have identified that daily self-report well-being questionnaires are sensitive to manipulations in training and competition load in high intensity intermittent team sport players [3,4,5]. The use of objective assessments to identify the maladaptive response associated with non-functional overreaching (NFOR) has received considerable attention [1,2]. Popular objective methods include countermovement jumps (CMJ) [1], resting HR (HR_rest_) and heart rate variability (HRV) [6]. However, the sensitivity of these measures to assess athlete recovery remains equivocal with some studies reporting a sensitivity or lack of sensitivity to changes in training load [2,7].

Although sport teams often train together, as individuals the time-course of recovery from a physical training stimulus is dependent on numerous factors including initial level of fitness, genetics, prior recovery and training exposure [8]. Therefore it is important that training and recovery metrics are considered on an individual basis rather than just in relation to the training group. Hence, establishing an individual baseline, the error within the measure and what constitutes a meaningful change [9] is important to effective monitoring enabling practitioners to identify red flags and modify training for each individual [10]. Furthermore, the utility of subjective and objective assessments in a team sport setting is dependent on the assessment’s ability to provide cost efficient, simple and accessible information on the athlete’s time-course of recovery [11].

To date, no study has communicated how subjective and objective assessments respond on a group and individual basis to known high and low external training loads. Therefore, the aim of this study is to compare the sensitivity of selected subjective and objective monitoring assessments in detecting changes in group and individual responses to a low load and high load bout of high intensity intermittent exercise.

## 2. Methods

### 2.1. Participants

Thirteen Rugby Union players from an academy team competing in the Association of Colleges Midland Elite League (mean ± SD: age 18 ± 1 years, stature 179 ± 6 cm, body mass 81.6 ± 18.6 kg) volunteered and provided informed consent for the study. Any participant who reported an injury during the study was excluded from any analysis. The study was undertaken during an in-season period. The participants’ normal weekly training involved three to four pitch based Rugby sessions (120 min per session), 1–2 gym based sessions (45 min per session) plus an 80 min competitive match. The study was approved by an institutional Ethics Committee (P20975) and conformed to the declaration of Helsinki.

### 2.2. Study Design

Three weeks prior to the commencement of the study, reliability of the WQ, CMJ, and HR indices was established. All subjective and objective assessments were made on three occasions at 9.00 a.m., during a five day period during a week where no training was undertaken and there was no competitive match (*n* = 13). Participants did not take part in any structured recovery methods during this period.

**Figure 1:**
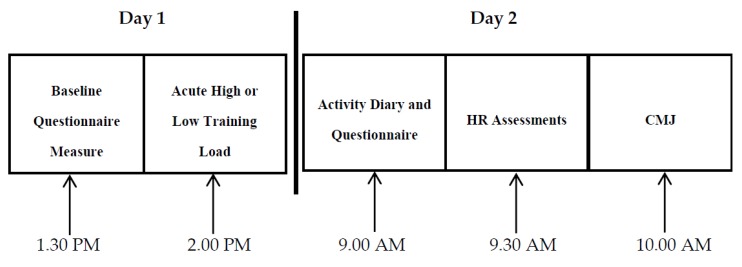
Schedule of each trial and subsequent objective and subjective assessments.

Using a counterbalanced crossover design, participants (*n* = 10, mean ± SD: age 18 ± 1 years, stature 180 ± 7 cm, body mass 86.6 ± 18.5 kg, estimated $\dot{V}O_{2}$ max 48 ± 4 mL·kg·bm^−1^) were assigned to a low load and a high load trial one week apart. Training load was manipulated by altering the duration of the LIST with the participant performing the Loughborough Intermittent Shuttle Test (LIST) [12] for 15 min (low load) or 90 min (high load). On the day following each trial (20 h post), subjective and objective monitoring assessments were carried out. Monitoring assessments included subjective (well-being questionnaire (WQ)) [3] and objective (countermovement jumps (CMJ), resting HR (HR_rest_) and resting heart rate variability (HRV)) measures. The participants carried out one LIST familiarisation (2 × 15 min blocks) two weeks prior to the study. To ensure consistency participants were familiarised with all monitoring assessments on a minimum of four occasions prior to undertaking the study. All testing was undertaken in a familiar environment where regular training and testing took place. Participants did not take part in any training in the week prior to or the between the trials. In addition, participants did not take part in any structured recovery methods during this period. The LIST and the CMJ were performed in an indoor sports hall. Measures of HR were taken in the changing rooms. Each trial and subsequent monitoring assessment was carried out at the same time each day (Figure 1) to avoid circadian variation.

The study was conducted during low load training weeks where only one light session with a technical emphasis was completed and there were no competitive matches. In the seven days preceding each trial participants were asked to refrain from carrying out any of their own additional training. In an attempt to quantify any additional training participants were requested to complete an activity diary on a daily basis. Seven participants reported carrying out 1–3 additional upper body strength training sessions per week. These were at similar time points prior to the high load and low load trials. No other additional training was reported. Participants were asked to wear the same footwear on each day, carry out their normal breakfast regime and abstain from caffeine 12 h prior to attending testing sessions.

### 2.3. Protocols

#### 2.3.1. Loughborough Intermittent Shuttle Test (LIST)

The LIST is a field based simulation designed to replicate the demands of intermittent team sports [12]. Participants were required to run at various speeds (sprinting, running, jogging and walking) determined by the group mean $\dot{V}O_{2}$ max in an indoor sports hall (mean ± SD: Temperature 17.4 ± 0.4 °C; Humidity 41.7 ± 5.3%). The group mean $\dot{V}O_{2}$ max was estimated using the Yo-Yo intermittent recovery test level 1 [13] carried out two weeks prior to commencing trials. One block of the LIST (Part A) was completed (15 min) for the low load trial. Six blocks of the LIST (90 min), with a three minute intermission between each block, were completed for the high load trial. Following each block of the LIST each participant gave a Rate of Perceived Exertion (RPE) using the CR-10 scale [14]. Training load (AU) was calculated using Session RPE (RPE × duration).

#### 2.3.2. Well-Being Questionnaire (WQ)

Questionnaires were completed immediately before and 20 h following each trial. Participants were asked to rate their perceptions of seven items each on a seven point scale (very good (+3), normal (0) to very poor (−3)) to monitor their perceptions of well-being related to: motivation to train, quality of previous night’s sleep, quality of recovery from previous day, appetite, feeling of fatigue, level of stress and level of muscle soreness [3]. Participants used a pen to complete a paper copy of the questionnaire on each occasion and did not discuss questionnaire responses with each other. For the purpose of analysis, the questionnaire items fatigue, stress and muscle soreness were reverse scored. Therefore, a higher score reflected greater fatigue, stress or muscle soreness.

#### 2.3.3. Resting Heart Rate (HR_Rest_) and Heart Rate Variability (HRV)

HR measures were determined via similar methods to those previously described [15]. Participants were positioned in a supine position for 10 min in the changing rooms (mean ± SD: Temperature 20.0 ± 1.1 °C; Humidity 40.0 ± 2.6%). It was requested that the participants stayed as still as possible, refrained from talking and remained as relaxed as possible. No attempt was made to control breathing rate and depth. A heart rate monitor (Team System, Suunto, Vantaa, Finland) was worn by participants across the chest and measured HR beat to beat. The lowest HR in the final five minutes was used to determine HR_Rest_. In addition, the final five min was analysed using Kubios software version 2.1 (University of Eastern Finland, Koupio, Finland). Time domain measures of HRV, the natural logarithm of the standard deviation of R-R intervals (ln SDNN) and the root square of the mean squared differences of successive R-R intervals (ln rMSSD) were determined.

#### 2.3.4. Countermovement Jump (CMJ)

All participants conducted a standardised 10 min warm up prior to the CMJ. The warm up involved a progressive increase in exercise intensity incorporating sport specific dynamic exercises (e.g., lunges, squats, kick throughs, skips, jumps) and running between two lines 20 m apart. CMJs were carried out in an indoor sports hall (mean ± SD: Temperature 21.5 ± 0.2 °C; Humidity 47.5 ± 2.4%). Following a set of three warm up jumps, participants carried out a total of six unloaded CMJ. The jumps were carried out in two sets of three jumps interspersed with 2–3 min recovery between sets. Jump time was measured as flight via a contact mat (Fusion Sport, Canberra, Australia). There was a three to five second intermission between each of the three jumps in each set. The participant was instructed to attempt to jump as high as possible. The best jump was recorded. No information regarding jump technique was given. However jumps were disqualified if: either (1) a participant pulled their thighs up to their chest to extend their flight time; or (2) both feet did not land back on the jump mat. If a jump was disqualified, corrective feedback was given and the participant performed another jump. If corrective feedback was provided, a longer intermission of 15–20 s was required between jumps.

### 2.4. Statistical Analysis

Internal consistency of the WQ was assessed using Cronbach α. Within-participant variation was expressed as typical error (TE) and calculated for CMJ and HR indices using the SD of the change in mean scores as described by Taylor, et al. [16]. TE was reported in absolute units and as a coefficient of variation (CV). The smallest worthwhile change (SWC) was set as 0.2 of between participant standard deviation [9].

For group analysis the data was examined via the Wilk’s Shapiro normality test. Paired *t*-tests were used to determine any differences between the low load and high load trials in normally distributed data. Non-normally distributed data including Session RPE and the subjective responses in the WQ were assessed using a bootstrapped paired *t*-test of 1000 replications [3]. Baseline WQ values prior to the low load and high load trials were compared. Pre to Post delta values from each trial were used to determine differences between WQ responses in the high load and low load trials. The uncertainty in the point estimate were reported as 95% confidence intervals. Effect sizes were reported using Cohen’s *d* (Trivial 0.00–0.19, small 0.20–0.59, moderate 0.60–1.19, large 1.20–1.99, very large >2.00) [17].

To determine individual responses to the high load and low load trials, the likelihood of a change for each individual was assessed considering the TE and the SWC. The likelihood of change is presented as percentage probabilities with a qualitative descriptor and any changes greater than 75% were considered substantial [9,18].

## 3. Results

### 3.1. Reliability Data

Internal consistency for the WQ ranged from 0.62–0.93 α (Table 1). Reliability expressed as a coefficient of variation for objective assessments was 5.2% for CMJ, 6.0% for HR_rest_, 4.9% for ln SDNN, 8.7% for rMSSD, (Table 1).

### 3.2. Group Responses

Session RPE was greater in the high load compared to low load (474 ± 187 AU, Table 2). Trivial to small differences were observed for items of the WQ between the two baseline measures (*d* = 0.1 to 0.5, *p* = 0.19–0.82)*.* Pre to post delta values were lower by a large extent for perceptions of sleep quality and of recovery following the high load compared to low load (−1.0 ± 1.1 AU vs. −0.3 ± 1.1 AU and −2.4 ± 1.8 AU vs. −0.2 ± 1.7 AU respectively, Table 2). Pre to post delta values for perceptions of motivation were moderately lower following the high load compared to low load (−1.9 ± 1.9 AU vs. −0.7 ± 1.7 AU, Table 2). Pre to post delta values for perceptions of muscle soreness were moderately higher following the high load compared to low load (2.0 ± 1.7 AU vs. 1.1 ± 1.5 AU, Table 2). Pre to post delta values were higher by a small extent for perceptions of appetite, fatigue and stress following the high load compared to low load (Table 2).

No differences in peak CMJ height were observed between high load and low load. A very large increase in mean HR_Rest_ was evident following the high load compared to low load (6 ± 4 b·min^−1^, Table 3). Moderate decreases in indices of HRV were observed following the high load compared to low load (−0.08 ± 0.08 ms, and −0.13 ± 0.08 ms, for ln SDNN and ln rMSSD respectively, Table 3).

### 3.3. Individual Responses

Training load, as indicated by Session RPE, ranged from 15 to 105 AU in low load compared to 240 to 810 AU in high load (Table 4). The majority of participants showed poorer perceptions of well-being following the high load compared to low load trials; with 7/9, 7/9, 6/9, 6/9, 5/9, 3/9 and 1/9 participants pre to post delta values for perceptions of recovery, sleep quality, motivation, muscle soreness, fatigue, stress and appetite deteriorating (Table 5). Only participant I reported WQ items that did not generally deteriorate following high load compared to low load. No participant had a substantial chance that the high load had a negative effect on CMJ performance compared to low load (5–72%, Table 6). Four individual participants (A, B, F, G) had a substantially higher mean HR_Rest_ following the high load compared to low load (76% to 91%, Table 7). Participants A and B yielded a substantially lower ln SDNN following the high load compared to low load (80% and 91%, respectively Table 8). In addition, participant B showed a substantially lower ln rMSSD in high load compared to low load (82%, Table 9). All other participants showed no substantial likelihood of change in HRV indices. Participants estimated $\dot{V}O_{2}$ max values ranged from 39 to 52 mL kg min^−1^. Participants A, F and I had the largest and participant G had the smallest estimated $\dot{V}O_{2}$ max values (Table 4).

## 4. Discussion

The main finding of the present study were that group responses showed selected items of the WQ (motivation, recovery, sleep quality and muscle soreness), HR_rest_ and indices of HRV were sensitive to changes in training load. However, in the current study the CMJ was not sensitive to acute fluctuations in training load. Individual WQ responses revealed 7/9, 7/9, 6/9, 6/9, 5/9, 3/9 and 1/9 participants reported deteriorations in perceptions of recovery, sleep quality, motivation, muscle soreness, fatigue, stress and appetite, respectively following high load compared to low load. Also, 4/6, 2/6 and 1/6 individuals for HR_rest_, ln SDNN and ln rMSSD, respectively, reported a substantial chance of a negative response after high load compared to low load.

This study indicates that selected WQ items could provide important information on the recovery status of a player given their sensitivity to changes in high load and low load. Moderate to large deteriorations in perceptions of motivation, recovery, sleep quality and muscle soreness were evident following the high load compared to low load. Previously the WQ identified poorer perceptions of well-being in elite youth soccer players in the later seasonal training blocks, potentially due to an accumulation of training load [3]. In addition, other self-report questionnaires have shown sensitivity to changes in training load across in-season training weeks assessed using three questionnaire items (fatigue, sleep quality and muscles soreness) in elite soccer players [19]. Furthermore, Gastin, et al. [5] reported items of wellness (fatigue, muscle strain, hamstring strain, pain/stiffness, power, sleep quality, stress and well-being) improved on a daily basis throughout the week following a high competitive match load and subsequent lower loads throughout the week in Australian rules football players.

These previous studies provide an insight into the ecological validity of the use of self-report questionnaires in team sports. However, other stresses accumulated during these periods in addition to the training load reported such as additional training and non-sport specific stress may have contributed to the proposed dose-response relationship between perceptions of well-being and training load. In the present study, the high load and low load trials were carried out in a controlled low load training week. Therefore, the present study highlights the sensitivity of daily subjective self-report questionnaires to changes in recovery status induced by training load manipulations.

The WQ items fatigue, stress and appetite were not sensitive to the differing high load and low load trials. In contrast, a previous study reported fatigue and stress were sensitive to within training week variation in high load and low load [5]. One factor influencing these differences could be the magnitude of stress in the present study may have been lower given match simulations may not elicit physiological responses as high as for a competitive fixture [20]. Conversely, given the relative isolation of high load and low load in the present study, fatigue, stress and appetite may be sensitive to accumulated loads but not high acute loads.

The individual differences presented in this study highlight individual responses to a fixed bout of high intensity intermittent exercise. Participant I had the lowest RPE load and one of the highest estimated $\dot{V}O_{2}$ max values. Therefore, the relatively lower internal load could in part explain the lack of any changes in perceptions of well-being in the WQ for participant I. Conversely participants A and F reported poorer perceptions of well-being despite similar $\dot{V}O_{2}$ max values. This highlights confounding factors in addition to training load such as relationships and lifestyle [21] which could influence perceptions of well-being during recovery. Hence, it is important each athlete is assessed on an individual basis.

Subjective measures have been reported to show greater sensitivity to increased acute and chronic training loads in comparison with objective measures [2]. The present study reported group CMJ performance was not sensitive to changes in training load. However, HR_Rest_ and HRV were sensitive to changes in acute training loads.

CMJ is a simple assessment which could be used as an objective measure of neuromuscular performance prior to training (Twist and Highton 2013). However, the present study suggests that the CMJ measure using a contact mat is not sensitive to high training loads. In contrast, previous studies show decrements in CMJ performance 24 h and 48 h following a competitive fixture (Ascensao et al. 2011; Fatouros et al. 2010; Magalhaes et al. 2010) and a 90 min match simulation (LIST; Bailey et al. 2007; Magalhaes et al. 2010). These differences could reflect difference in the magnitude of the acute load (De Hoyo et al. 2016; Magalhaes et al. 2010). Furthermore, more expensive equipment such as force plates may be required to detect neuromuscular fatigue in a CMJ (Gathercole et al. 2015).

Group analysis of HR indices in the present study suggests HR_Rest_ and HRV measures were sensitive to changes in training load. These measures of the autonomic nervous system have previously been proposed as a marker of NFOR and are reported to be sensitive to acute changes in training load [6,22]. Often team sport players are required to perform competitively twice a week [23]. Therefore, the sensitivity of HR indices to high acute training loads might be a useful tool for coaches and practitioners.

In an applied setting, monitoring must be carried out on an individual level due to the aforementioned individual differences. On an individual level it has been proposed that HR indices are too variable to assess athletes based upon a single measure (Buchheit 2014; Plews et al. 2013). Individual increases in 4/6 participants were evident for HR_rest_, but only 2/6 and 1/6 participants reported a reduction in ln SDNN and ln rMSSD, respectively. Given the magnitude of the ‘noise’ and the SWC in measures of HR, single infrequent assessments of HR indices may only be sensitive to very large fluctuations in training load. Therefore, frequent daily assessment of HR indices using a rolling average would be required to reduce the ‘noise’ of the measurement which is often not practical in team sport players (Buchheit 2014; Plews et al. 2013).

A limitation to the present study was the small sample size. The study population was a convenience sample recruited from a group of rugby players competing at a select level. A follow-up study with more participants is needed to confirm the findings of the present study.

## 5. Conclusions

Subjective questionnaire items are sensitive to acute changes in training load and can be used as a standalone measure to assess the time-course of recovery in team sport players in an applied setting. Items such as recovery, sleep quality, motivation and muscle soreness were most sensitive to changes in training load and should be considered in the subjective self-report of athletes. CMJ were not sensitive to changes in acute training load, but HR indices showed sensitivity to changes in acute training load when assessed on a group level. However, when assessing athletes on an individual level, the large day to day variation in HR indices lacked the sensitivity to detect small meaningful changes. Given the time cost of data collection and analysis and the lack of sensitivity to individual changes, daily monitoring using objective assessments may not practical be in team sports. Therefore, daily subjective assessments may provide greater utility in an applied setting compared with objective assessments.

## Figures and Tables

**Table 1:** Day to day reliability of well-being questionnaire (WQ) items, countermovement jump (CMJ) and heart rate (HR) indices.

Item	Trial 1 Mean	Trial 2 Mean	Trial 3 Mean	Cronbach α	TE (CV)	SWC (CV)
Motivation (AU)	1.00 ± 1.00	1.00 ± 1.22	1.23 ± 1.30	0.78	-	-
Sleep quality (AU)	1.23 ± 0.93	1.15 ± 1.41	0.54 ± 1.33	0.71	-	-
Recovery (AU)	0.31 ± 1.38	1.08 ± 1.19	1.08 ± 1.12	0.62	-	-
Appetite (AU)	1.08 ± 1.38	1.23 ± 1.17	1.31 ± 1.32	0.82	-	-
Fatigue (AU)	−0.08 ± 1.55	−0.08 ± 1.19	−0.62 ± 1.26	0.65	-	-
Stress (AU)	−0.08 ± 1.04	0.15 ± 1.72	−0.08 ± 1.44	0.93	-	-
Muscle soreness (AU)	0.00 ± 1.63	−0.77 ± 1.42	−0.69 ± 1.38	0.77	-	-
CMJ (cm)	38.6 ± 6.4	39.0 ± 5.5	36.9 ± 5.7	-	2.0 (5.2)	1.5 (3.9)
HR_Rest_ (b·min^−1^)	65 ± 6	68 ± 7	68 ± 7	-	4 (6.0)	2 (3.0)
ln SDNN (ms)	1.82 ± 0.19	1.84 ± 0.15	1.88 ± 0.17	-	0.09 (4.9)	0.04 (2.2)
ln rMSSD (ms)	1.73 ± 0.29	1.70 ± 0.30	1.76 ± 0.30	-	0.15 (8.7)	0.08 (4.6)

Data expressed as mean ± SD. Internal consistency of WQ items (motivation, sleep quality, recovery, appetite, fatigue stress and muscle soreness) assessed using Cronbach’s α. Typical error of measurement (TE) expressed as a coefficient of variation (CV) is given for counter movement jump (CMJ), mean resting HR (HR_Rest_), the natural logarithm of the standard deviation of R-R intervals (ln SDNN) and the natural logarithm of the root square of the mean squared differences of successive R-R intervals (ln rMSSD). The SWC is also presented to allow comparison with typical variations (*n* = 13).

**Table 2:** Comparison between the effects of high load and low load trials on Session Rate of Perceived Exertion (RPE) during Loughborough Intermittent Shuttle Test (LIST) and subsequent WQ responses.

	Low Load	High Load	Change	Confidence Interval	*p* Value	Cohens *d*	Effect Size
Session RPE (AU)	47 ± 33	521 ± 174	474 ± 187	369 to 579	<0.01	3.8	very large
Motivation (AU)	−0.7 ± 1.7	−1.9 ± 1.9	−1.2 ± 1.8	−2.2 to −0.1	0.12	0.7	moderate
Sleep quality (AU)	0.3 ± 1.1	−1.0 ± 1.1	−1.3 ± 1.5	−2.3 to −0.6	0.12	1.2	large
Recovery (AU)	−0.2 ± 1.7	−2.4 ± 1.8	−2.2 ± 2.4	−3.6 to −0.7	0.03	1.5	large
Appetite (AU)	0.0 ± 1.7	0.7 ± 0.9	0.7 ± 2.1	−0.2 to 2.1	0.38	0.5	small
Fatigue (AU)	0.2 ± 1.6	0.9 ± 1.6	0.7 ± 2.3	−0.8 to 1.9	0.41	0.4	small
Stress (AU)	0.2 ± 0.2	0.6 ± 1.6	0.3 ± 1.7	−0.7 to 1.4	0.57	0.4	small
Muscle soreness (AU)	1.1 ± 1.5	2.0 ± 1.7	0.9 ± 2.8	−0.8 to 2.6	0.36	0.6	moderate

Mean ± SD, 95% confidence intervals, *p* value, t-statistic and effect size for Session RPE and pre to post trial delta values in both the high load and low load trials for perceptions of motivation, sleep quality, recovery, appetite, fatigue, stress and muscle soreness. Mean change ± SD reported as a delta value from the high load to low load trial (*n* = 9).

**Table 3:** Peak CMJ performance and indices of HR at rest following the high load and low load trials.

	Peak CMJ Height (cm)	Mean HR_Rest_ (b·min^−1^)	ln SDNN (ms)	ln rMSSD (ms)
Low Load	37.2 ± 4.4	58 ± 1	1.96 ± 0.09	1.94 ± 0.18
High Load	37.2 ± 4.4	64 ± 4	1.88 ± 0.13	1.81 ± 0.18
Mean Change	0 ± 1.8	6 ± 4	−0.08 ± 0.08	−0.13 ± 0.08
Confidence Interval	−1.3 to 1.3	1 to 10	−0.18 to 0.00	−0.21 to −0.04
t-Statistic	−0.04	3.28	−2.64	−3.70
*p* value	0.97	0.02	0.04	0.01
Cohens *d*	0.0	2.1	0.7	0.7
Effect Size	trivial	very large	moderate	moderate

Mean ± SD, 95% confidence intervals, *p* value, t-statistic and effect size for countermovement jump (CMJ) (*n* = 10) mean resting HR (HR_Rest_) (*n* = 6), the natural logarithm of: the standard deviation of R-R intervals (ln SDNN) (*n* = 6) and the root square of the mean squared differences of successive R-R intervals (ln rMSSD) (*n* = 6).

**Table 4:** Estimated $\dot{V}O_{2}$ max values and Session RPE during LIST in high load and low load trials for individual participants.

Participant	Estimated $\dot{V}O_{2}$ Max (mL kg bm^−1^)	Session RPE (AU)	
		Low Load Trial	High Load Trial
A	52	60	420
B	-	75	405
C	50	15	525
D	48	75	240
E	49	15	750
F	51	15	585
G	39	60	810
H	47	30	600
I	51	15	375
J	45	105	495

Data expressed as absolute individual scores for $\dot{V}O_{2}$ max and Session RPE (*n* = 10).

**Table 5:** Individual differences in WQ responses between the high load and low load trials.

Participant	Motivation	Sleep Quality	Recovery	Appetite	Fatigue	Stress	Muscle Soreness
A	−2	−1	−3	0	0	0	−1
B	2	−1	−1	−1	−4	0	−4
C	−2	−1	0	0	3	4	1
D	−3	−2	−4	0	2	0	−1
E	−3	−5	−6	1	4	1	6
F	−2	−1	−1	0	1	0	2
G	1	0	−3	6	0	−1	1
H	−2	−1	−4	0	1	1	3
I	0	0	2	0	−1	−2	1

Perceptions of motivation, sleep quality, recovery, appetite, fatigue, stress and muscle soreness calculated as a pre to post trial delta value in both the high load and low load trials. Data presented as a change score between the high load and low load trial delta values (*n* = 9).

**Table 6:** Individual responses following high load and low load trials for peak jump height measured during a CMJ.

Participant	Low Load (cm)	High Load (cm)	Change (cm)	Likelihood of Effect (%)			Qualitative Descriptor
				−ve	Trivial	+ve	
A	40.3	38.6	−1.7	53	34	13	possibly, may not be −ve
B	36.0	32.8	−3.2	72	22	5	possibly, may not be −ve
C	32.3	33.4	1.1	18	37	44	unlikely, probably not −ve
D	37.2	39.0	1.8	13	33	54	unlikely, probably not −ve
E	44.7	43.9	−0.8	40	38	21	possibly, may not be −ve
F	41.5	41.0	−0.5	36	39	24	possibly, may not be −ve
G	30.5	31.0	0.5	24	39	26	unlikely, probably not −ve
H	33.2	32.5	−0.7	39	39	22	possibly, may not be −ve
I	39.0	39.1	0.1	29	40	31	possibly, may not be −ve
J	37.1	40.3	3.2	5	22	72	unlikely, probably not −ve

Data presented as absolute scores, delta values, percentage of likelihood of change (negative (−ve), trivial, positive (+ve)) and qualitative descriptor (*n* = 10). Smallest worthwhile change (SWC) and Typical Error (TE) from reliability data (Table 1) used to determine likely limits. SWC = 1.5 cm and TE = 2.0 cm.

**Table 7:** Individual responses following high load and low load trials for mean resting HR.

Participant	Low Load (b·min^−1^)	High Load (b·min^−1^)	Change (b·min^−1^)	Likelihood of Effect (%)			Qualitative Descriptor
				−ve	Trivial	+ve	
A	59	69	10	91	6	2	likely, probably higher
B	57	66	9	89	8	3	likely, probably higher
C	59	60	1	43	27	30	possibly, may not be higher
F	57	65	8	85	10	4	likely, probably higher
G	56	62	6	76	16	9	likely, probably higher
I	59	59	0	36	27	36	possibly, may not be higher

Data presented as absolute scores, delta values, percentage of likelihood of change (negative (−ve), trivial, positive (+ve)) and qualitative descriptor (*n* = 6). Smallest worthwhile change (SWC) and Typical Error (TE) from reliability data (Table 1) used to determine likely limits. SWC = 2 b·min^−1^ and TE = 4 b·min^−1^.

**Table 8:** Individual responses following high load and low load trials for the natural logarithm of the standard deviation of normal to normal intervals (ln SDNN).

Participant	Low Load (ms)	High Load (ms)	Change (ms)	Likelihood of Effect (%)			Qualitative Descriptor
				−ve	Trivial	+ve	
A	1.88	1.66	−0.22	91	6	3	likely, probably lower
B	2.00	1.85	−0.15	80	12	7	likely, probably lower
C	1.91	1.89	−0.02	44	24	32	possibly, may not be lower
F	1.88	1.84	−0.04	50	23	27	possibly, may not be lower
G	2.02	1.99	−0.03	47	24	29	possibly, may not be lower
I	2.09	2.03	−0.06	56	22	22	possibly, may not be lower

Data presented as absolute scores, delta values, percentage of likelihood of change (negative (−ve), trivial, positive (+ve)) and qualitative descriptor (*n* = 6). Smallest worthwhile change (SWC) and Typical Error (TE) from reliability data (Table 1) used to determine likely limits. SWC = 0.04 ms and TE = 0.09 ms.

**Table 9:** Individual responses following high load and low load trials for the natural logarithm of the root square of the mean squared differences of successive R-R intervals (ln rMSSD).

Participant	Low Load (ms)	High Load (ms)	Change (ms)	Likelihood of Effect (%)			Qualitative Descriptor
				−ve	Trivial	+ve	
A	1.66	1.51	−0.15	63	23	14	possibly, may not be lower
B	2.09	1.81	−0.28	82	13	5	likely, probably lower
C	2.01	1.96	−0.05	44	28	27	possibly, may not be lower
F	1.79	1.73	−0.06	46	28	26	possibly, may not be lower
G	2.09	1.97	−0.12	57	25	18	possibly, may not be lower
I	2.01	1.91	−0.10	54	26	20	possibly, may not be lower

Data presented as absolute scores, delta values, percentage of likelihood of change (negative (−ve), trivial, positive (+ve)) and qualitative descriptor (*n* = 6). Smallest worthwhile change (SWC) and Typical Error (TE) from reliability data (Table 1) used to determine likely limits. SWC = 0.08 ms and TE = 0.15 ms.
